# Abandonment landscapes: user attitudes, alternative futures and land management in Castro Laboreiro, Portugal

**DOI:** 10.1007/s10113-018-1294-x

**Published:** 2018-02-10

**Authors:** Emma H. van der Zanden, Sónia M. Carvalho-Ribeiro, Peter H. Verburg

**Affiliations:** 1 Environmental Geography group, Institute for Environmental Studies (IVM), VU University Amsterdam, De Boelelaan 1087, 1081 HV Amsterdam, The Netherlands; 2 Theoretical Ecology Research Group, Faculdade de Ciências da Universidade de Lisboa, Lisbon, Portugal; 3 Present Address: Centro de Sensoriamento Remoto, Universidade Federal de Minas Gerais UFMG, Av. Antônio Carlos, 6627, Belo Horizonte, MG Brazil

**Keywords:** Agricultural abandonment, Portugal, Cultural heritage, Rewilding, Questionnaire, Landscape preferences

## Abstract

Land abandonment is an important process for the European Union, which primarily occurs in less productive, remote and mountainous areas with unfavourable conditions for agriculture. Future management directions of these abandonment areas are under debate, with increasing calls to adjust policies to the local characteristics, including the promotion of rewilding and the management of succession of larger areas of less-productive land. While there is an increase in studies focusing on the environmental impacts of land abandonment, there are few studies that focus on the perceptions of abandonment by different user groups, even though an understanding of local perceptions, opportunities and trade-offs associated with changing land management is crucial for landscape-related policies and planning measures. In a case study in Northern Portugal, we used a combination of statements, photograph rating exercises and open questions to assess the perceptions of local inhabitants, visitors and experts regarding land abandonment and their preferences of different possible trajectories after abandonment. The results show that all user groups have a negative response towards abandonment and associate it mainly with negative emotions and the loss of heritage and traditions. The assessment of the different abandonment stages and outcomes clearly yielded different preferences and explanations, which can be used as input for finding a common ground for landscape management, reducing conflict and as a starting point for a more spatially targeted and nuanced management approach.

**Electronic supplementary material:**

The online version of this article (10.1007/s10113-018-1294-x) contains supplementary material, which is available to authorized users.

## Introduction

European rural landscapes are currently undergoing large changes, in which land abandonment and agricultural decline is an important process, especially in mountainous areas (MacDonald et al. 2000; Soliva et al. 2008). The future management directions of abandonment areas are under debate. Currently, many agri-environmental subsidies target marginally productive land to maintain both low-intensity farming practices and (agro-)biodiversity conservation (EEA 2010). However, there are increasing calls for a reform of this system based on local characteristics, which include the promotion of rewilding and succession management of larger areas of less-productive land (Merckx and Pereira 2014).

An overview of recent studies on land abandonment in Europe showed that land abandonment primarily occurs in areas with unfavourable conditions for agriculture, often being remote and mountainous regions. Secondary drivers of land abandonment include rural depopulation and regional specific factors regarding land ownership and tax regimes (Keenleyside and Tucker 2010; Rey Benayas et al. 2007). Even with the support of subsidies such as the less favoured areas (LFA) support and agri-environmental payments, agriculture in these areas is often not competitive (Keenleyside and Tucker 2010).

Rewilding has been proposed as a new approach to manage abandoned farmlands in Europe to contribute to nature conservation objectives (Jepson 2015; Merckx and Pereira 2014; Navarro and Pereira 2012). However, abandoned land is often perceived negatively as unkempt land and associated with the loss of economic stability within a region by the general public (Bauer et al. 2009; Höchtl et al. 2005). Another challenge to rewilding as a management option is that it can lead to different long-term outcomes depending on biotic and abiotic conditions (Navarro and Pereira 2015). Furthermore, certain regions need assisted or active regeneration, e.g. depending on the native seed bank (Benayas and Bullock 2015; Munroe et al. 2013).

Planning and policy that aim at either preventing agricultural abandonment or managing abandoned areas should consider social and environmental challenges related to agricultural abandonment. A common method for the assessment of rural management options in landscape research focuses on public preferences of land use management and landscape aesthetics. However, studies that focus specifically on land abandonment and perceptions for its management are still few (e.g. Benjamin et al. 2007; Ruskule et al. 2013). Studies that focus on the perception of land abandonment among different societal groups are also lacking (Hunziker et al. 2008). This is an important aspect in the case of land abandonment, since the users of landscapes are no longer only farmers, but now often also include tourists and other visitors. This is especially so for areas facing abandonment, which often have many scenic elements. Knowledge about perceptions by different societal groups is crucial for landscape-related policies and planning measures, e.g. supporting the identification of planning goals that reconcile the views of various public groups and minimize conflicts (Hunziker et al. 2008; Smith et al. 2011).

Therefore, the aim of this paper is to assess how different landscape user groups experience the process of land abandonment in a study area with ongoing land abandonment. In this research, we focus both on the perception of the overall impact of land abandonment and on the public preferences of different groups for different “abandonment landscapes”. These abandonment landscapes represent both the environmental outcome, the stage of abandonment and regrowth and different possible trajectories after abandonment. Using a combination of statements, photograph rating exercises and open questions, we aim to move beyond a one-dimensional assessment of abandonment. Based on the results from the questionnaires and interviews, we explore new directions for future management strategies.

We hypothesize that the general attitude towards abandonment will be negative and linked to negative emotions, especially among local inhabitants (Benjamin et al. 2007; Höchtl et al. 2005; Ruskule et al. 2013). However, we expect visitors to be less negative. For experts, we believe that their main focus is on a return to traditional cultural landscapes (Hunziker et al. 2008). We further expect that the assessment of abandonment landscapes and trade-offs will give a more nuanced view, including possible benefits related to abandonment.

This research is located in the Portuguese parish of Castro Laboreiro, Northern Portugal, which is part of the Peneda-Gerês National Park. This area has undergone widespread abandonment during the past 60 years and faces related economic, environmental and social issues. This area can be taken as an example of the areas that experience loss of traditional mountain farming systems, which are threatened throughout Europe as a consequence of agricultural abandonment (see Beilin et al. 2014; Lomba et al. 2014).

## Methods

### Case study area

**Fig. 1:**
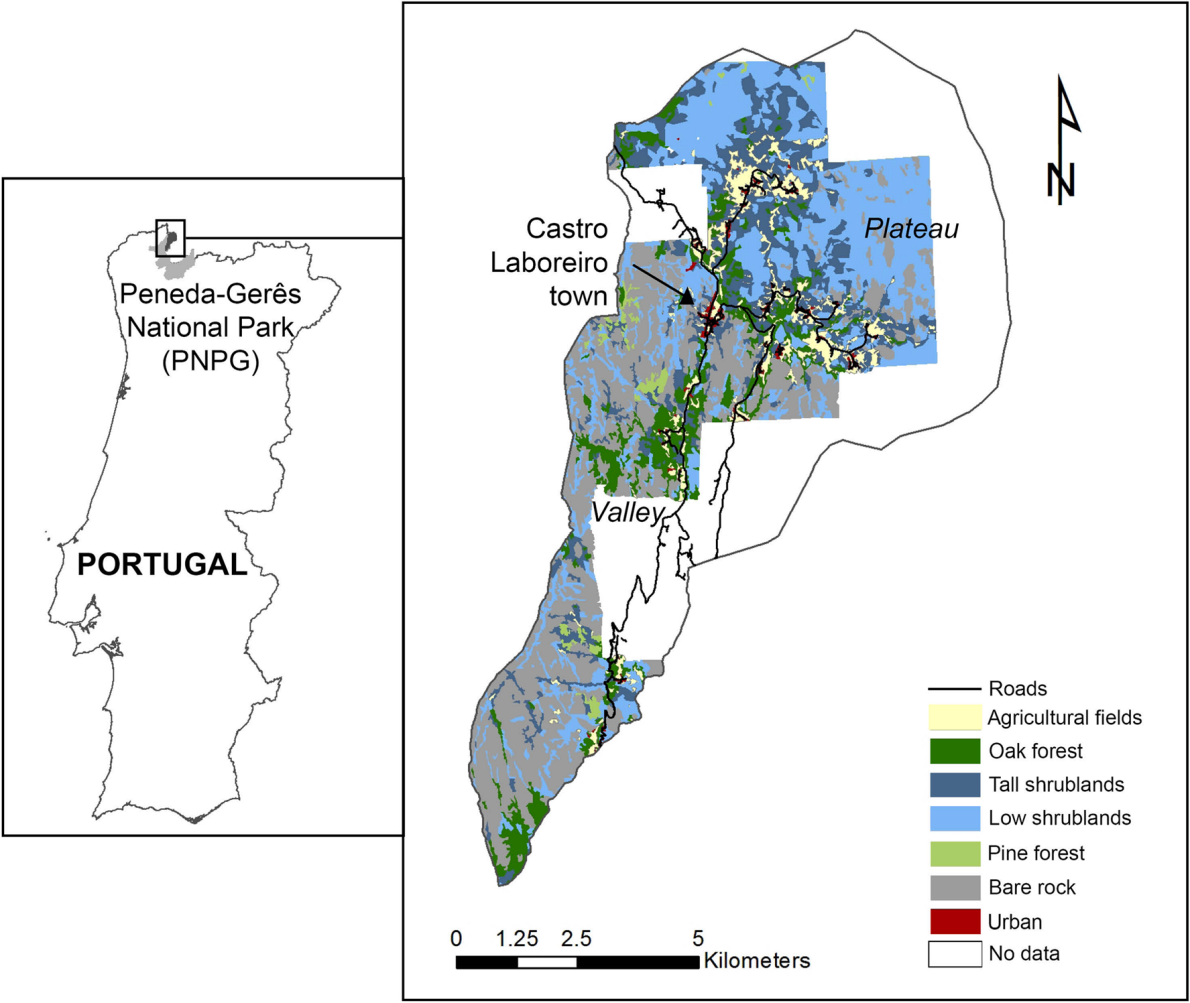
Location of the Castro Laboreiro case study area, land use based on 2007 aerial georeferenced photographs (Rodrigues 2010)

The Parish of Castro Laboreiro (9940 ha) is part of the municipality of Melgaço. The relief divides the area in a plateau and a valley section from north to south (Fig. 1). The elevation ranges between 300 and 1340 m above sea level. The area has a temperate Mediterranean climate, with mild winters and warm summer months. Since 1971, Castro Laboreiro is part of the Peneda-Gerês National Park with the aim to protect the high natural value landscape. It is also part of Natura 2000, a European protected area network. The park authority has considerable influence on planning and development in the area, including national park stipulations for development plans, residential zoning, architectural specifications and landscape management (e.g. van Berkel et al. 2011).

Castro Laboreiro underwent large land use changes over the past 60 years. Historically, agro-pastoral activities were widespread, with seasonal migration from summer villages at the plateau (*brandas*, 15 in total) to winter villages in the valley (*inverneiras*, 18 in total). Agriculture consisted of small-scale farms (< 2 ha) with grazing on common land (*baldios*) (Domingues and Rodrigues 2008). Land abandonment first occurred as a result of political and socio-economic changes in the 1940s. Reduced agricultural incomes led to male out-migration between the 1960s and 1980s. While many former inhabitants returned in the 1980s, many discontinued their practice in the early 1990s as a result of diminishing government support and adaptive capacity (Edwards and Fernandes 1999).

As a result of strong depopulation, the parish is now inhabited by an ageing, predominantly female, population of 540 residents (Instituto Nacional de Estatistica 2012). The seasonal transhumance has decreased since the early 1980s and many farmers chose to stay in the well-established plateau houses, with the plateau mostly used for grazing cattle (*cachena*, *barrosã*), sheep and goats. In the valley, homes and fields are increasingly abandoned (Domingues and Rodrigues 2008). Remaining farmers receive subsidies to maintain traditional farming and pastoral activities. The number of newcomers has increased, whom mainly focus on the tourist sector or own hobby farms or vacation homes. As a result of these major demographic and land use changes, the main policy concern for the area is how the agricultural abandonment will affect the regional aesthetic character of the area, possibly reducing the tourist attraction (van Berkel et al. 2011).

### Questionnaire setup

Our research method was based on a structured questionnaire consisting of six blocks, each having a different purpose within the study. Three different versions of the questionnaire were used, with specific questions targeted to local inhabitants, visitors and experts. We chose to use multiple methods within the questionnaire, including both qualitative open-ended questions as well as questions suited for quantitative analysis and a photo ranking exercise to assess the different dimensions of abandonment. Most questions were cross-checked in the questionnaire to ensure robustness of results. To ensure a correct phrasing and understanding of the questions, we pre-tested the questionnaire. See Online Resource 1 for a detailed setup of the questionnaire.

The first section of the questionnaire was used to assess the current situation, starting with a visual description: “If you could take a photo of the aspects you like the most about the Castro Laboreiro countryside, what would you photograph, and where?”. This question was framed in this way to generate responses that focus on the aspects of the landscape that respondents would convey to others, following Carvalho-Ribeiro et al. (2013). The second section focused on different dimensions of land abandonment. Among others, we were interested in how respondents experience the trade-off between the different positive and negative consequences of land abandonment in the area.

**Fig. 2:**
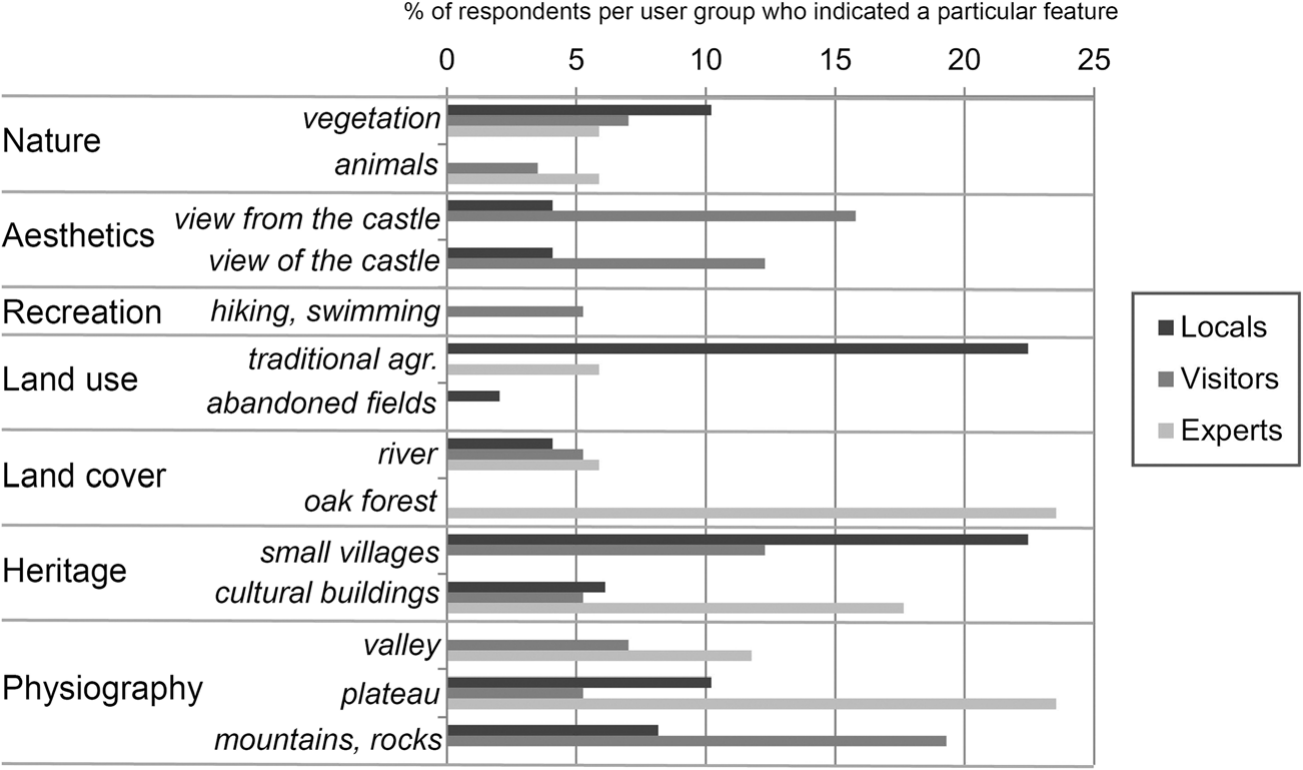
Categorized responses of the most liked aspects of the Castro Laboreiro countryside

**Fig. 3:**
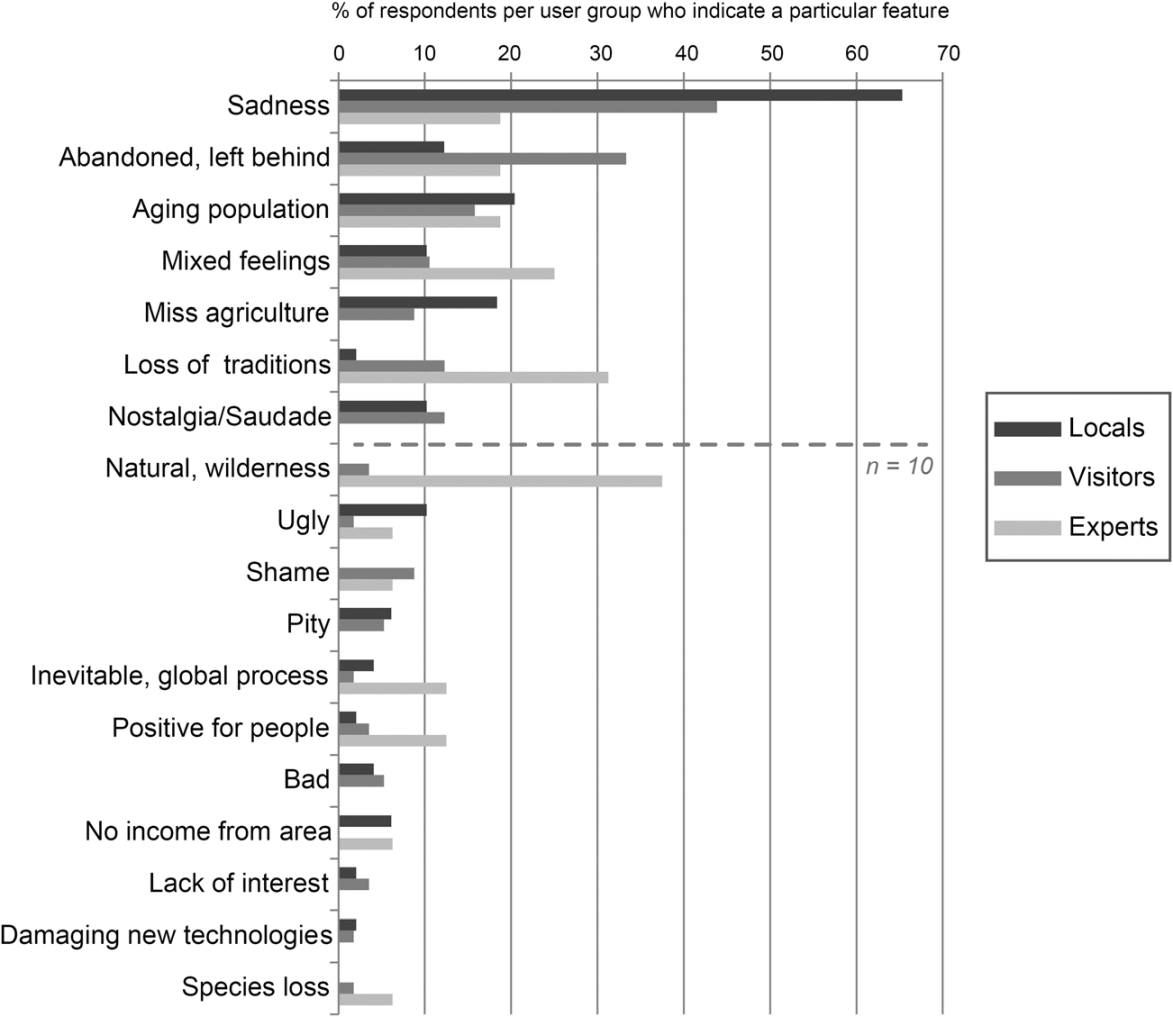
Words and emotions related to discontinuing farming traditions in Castro Laboreiro (percentage of user groups who indicated a particular feature). Only the group differences for “sadness” and “loss of traditions” are significant (Fisher’s exact test and post-hoc analysis; *p* = 0.002 and *p* = 0.005, respectively)

The third section consisted of a photograph ranking exercise regarding the attractiveness of the landscape. Each respondent was shown pairwise photographs (close-up versus distant view) representing different growth stages of typical abandonment landscapes in the area, which were identified in the field and discussed with ecologists familiar with the area. In the exercise, respondents ranked the photographs from most attractive (1/5) to least attractive (4/5). Four common abandonment landscapes were identified for the area: oak forest, tall shrublands, low shrublands and Acacia encroachment (see Online Resource material 1 Figs. 2 and 3; Online Resource 1 also provides information on the main species composition). We did separate ranking exercises for the plateau and the valley, because of the different landscapes and traditional function. For the valley, a fifth category was included (plantations), which do not exist on the plateau. We used some digital post-processing to ensure comparable size, contrast, colouring and view depth of the pictures. The use of photographs in landscape preference studies is a common practice, as photos show the landscape in a holistic way and close to a real-life experience of the landscape (see, e.g. Palmer and Hoffman 2001).

In the final sections of the questionnaire, we focused on different management strategies, as well as on specific user group characteristics and the socio-economic profile of the respondents. The management strategies were addressed using questions on the preferred management outcome, preference for specific future management practices and the actors with the largest responsibility for future management of abandonment areas. The socio-economic section was based on a number of personal variables that are expected to influence the preference of individuals towards wild versus managed natural settings, as we believe this to be an important influence in an abandonment situation. Although there are no firm conclusions about underlying variables (Kaplan and Kaplan 1989), variables that are expected to be influential are, among others, place of residence, socio-economic characteristics, connection to agriculture, environmental attitude and recreational motives. The degree of human influence needed to manage a landscape is also a key dimension that helps in describing people’s landscape perception (Van den Berg and Koole 2006).

### Sampling approach

A theoretical sampling approach was applied, which is also used in comparable studies (Hunziker 1995; Hunziker et al. 2008; Ruskule et al. 2013). This approach samples for a maximum variety among the respondents according to sample-selection criteria relevant to the particular objectives of the study. See Online Resource 1 for the specific criteria per user group.

Using the questionnaire, we sampled a total of 122 persons (49 classified as being local inhabitants, 57 as visitors and 17 as experts). Each questionnaire took approximately 45 min to complete, although the interviews with local inhabitants and experts often exceeded this. The questionnaires were conducted in Portuguese and English in August–September 2014, by a research team consisting of two Portuguese and one Dutch researcher. Both the Portuguese and English questionnaires had been pre-tested to ensure consistency in terminology and meaning.

### Data analysis

The questionnaire was coded in a database. Open questions were first transcribed into full text, and then the most commonly used phrases and keywords were listed and grouped, using the keywords in context analysis (KWIC) technique (Ryan and Bernard 2003). For the statistical analysis, the data were first tested for normality and homogeneity of variance. We used the non-parametric Kruskal-Wallis test followed by a Bonferroni-Dunn test to analyse the group differences of the ordinal responses. For binary data, we used Pearson’s chi-squared test and Fisher’s exact test, with the latter being more suitable for small sample sizes. We used linear regression to fit a model to explain the overall abandonment impact from the socio-economic variables describing the respondents. For variable selection, we selected the best model from the regression dataset by performing a pre-test with all possible combinations, followed by a backward stepwise selection. For the selection of the models, we used a critical level of *α* = 0.01 and Akaike’s information criterion (AIC), in which we used the variance inflation factor (VIF) and removed variables with a correlation > 0.7 to avoid multicollinearity. Spearman’s rank correlation coefficient was used to check for correlations between explanatory variables. All models were implemented in R (R Development Core Team 2008).

## Results

### Profile of the different user groups

The average age of respondents was 47 years, with local respondents having a higher average of 53 years (see Online Resource 2 for descriptive statistics). Our sample consists of slightly more females than males, especially for local inhabitants and experts. These results were expected for local inhabitants, as Castro Laboreiro inhabitants are mainly women (62%) and elderly, with most in the 65–69 years age category (Instituto Nacional de Estatistica 2012). The respondents generally had a high education level, especially among visitors. The locations of origin, as expected, differ per user group. The vast majority of local inhabitants (78%) originated from Castro Laboreiro. Less of the expert group originated from the region, although all were familiar with Castro Laboreiro and the Peneda-Gerês National Park. This group included varied occupations related to land management, such as local and regional policy makers (Ministry of Agriculture), employees related to the National Park and researchers from different institutions.

Visitors of Castro Laboreiro had a varied profile, with the majority being a domestic tourist (75%). Time spent in the area is equally divided between first-time visitors and those visiting 2–5 times and > 10 times (each 32% of our sample). Almost half the visitors came to Castro Laboreiro on a daytrip, followed by those visiting for multiple days but less than a week (32%), with the remaining visiting for a week or longer. Individuals reported multiple purposes for their visit, of which the main ones were sightseeing (60%), followed by recreation (38%) and visiting family or friends (24%). Two visitors specifically mentioned a visit to see the local dog breed (*Cão de Castro Laboreiro*). Twenty-nine respondents clarified their recreation purposes, with most focusing on walking/hiking (27 respondents), followed by other outdoor sports (13 respondents) and a nature focus (11 respondents).

The profile of the local inhabitants highlights the traditional agricultural origin of Castro Laboreiro, with high percentages of inhabitants having a connection with agriculture and finding human influence necessary in natural areas. Although only a limited number of respondents list farming as their primary profession, 73% of all local respondents owned fields and/or animals (e.g. cattle, sheep, goats). Agriculture is mainly focused on home consumption and the majority of land and animal owners gain less than half their income from agriculture. Fifty-five percent of local inhabitants report that they make use village communal lands for livestock grazing. More than half of the land owners (65%) mentioned that they have abandoned (part of) their land, due to retirement, reduced physical ability linked with age, the lack of subsidies and the limited financial payback.

### Current situation and landscape preference

We analysed the current landscape preferences of the respondents by classifying the aspects of the Castro Laboreiro landscape that the respondents liked the most (Fig. 2). The majority of the respondents would take a photo of the small villages (heritage category), followed by the mountain landscape (physiography category) and the traditional agriculture (land use category). Traditional agriculture was mentioned significantly more often by local inhabitants than by the other user groups, when testing for all classes with an *n* above 10 (Fisher’s test *p =* 0.0001974, local—visitor). The view from the Castle (aesthetic category) and the mountain/rock landscape (physiography category) was mentioned most often by visitors, who mentioned significantly more aspects from aesthetic categories (Fisher’s test *p =* 0.003776). Oak forest (land cover category) was most often brought up by experts, as they mentioned significantly more aspects from the land cover categories than the other user groups (Fisher’s test *p =* 0.008502).

We also classified the aspects of the Castro Laboreiro landscapes that the respondents disliked the most. Here, the overall highest categories were modern houses disrespecting the traditional style (17%), abandoned villages (10%) and litter (10%), as well as signs of farming discontinuation (8%) such as stone walls falling apart or unkempt hedges. It should also be noted that more than 20 respondents said that they like everything in the area.

When comparing these preferences to the way that local inhabitants describe the “Castrejo way of being” to imaginary outsiders of the area, this shows a large overlap. Traditional agriculture is mentioned the most, often accompanied by an image of a women working in the fields wearing a traditional outfit, within a mountain setting. As land cover, oak forest was specifically mentioned, while reported heritage features included, e.g. pilgrimage places, the local dog breed and baking bread in a traditional way. When focusing on the local culture, the hard-working attitude, social cohesion and closed society, with some distrust to outsiders, were among the most mentioned aspects.

### Opinions on dimensions of land abandonment

#### Perception of land abandonment

Abandonment in the area of Castro Laboreiro has clear visual effects on the local landscape, with respondents reporting the increase of shrubland and specific shrub species, decrease of domestic animals, increase of alien species and the visible collapsing of houses and other structures (e.g. stone walls). Several respondents report on the increase of wild animals, with a number of local inhabitants discussing their negative attitude towards the wolf (Iberian wolf, *Canis lupus signatus*), which they perceive as dangerous and a threat to the domestic animals.

The overall impact of the discontinuation of traditional farming is perceived negatively among all user groups, although there are some differences between the groups. Experts give a significant more positive rating, 2.8 on a 5-point Likert scale ranging from very negative to very positive, compared to both local inhabitants and visitors (2.0 and 2.1, respectively), as revealed through a post-hoc Dunn’s test (*χ*^2^ = 9.2865, d.f. = 2, *p* = 0.01). There is no discernible difference between local inhabitants and visitors. Regression results indicate that respondents from the expert group are more likely to be positive about abandonment. Also, more positive attitudes to abandonment are found with those that indicate that human influence is not a necessary aspect of a natural area, those that are not from Castro Laboreiro or surroundings and those that have a connection with agriculture. The last two are, however, not significant (for details see Online Resource 2).

The overall negative attitude towards land abandonment is reflected in the words or emotions that respondents linked to abandonment, which is most often associated with words as sadness, (the feeling of being) abandoned and left behind and emotions related to the loss of a former agricultural lifestyle (e.g. nostalgia, *saudade*—the latter is a Portuguese term for a deep nostalgic of melancholic feeling) (see Fig. 3). A 54-year-old local inhabitant for instance notes: “Sadness, because we can’t work them [the fields] and our kids had to leave because they didn’t have a future here”. A smaller number (12% of all respondents) reported mixed or more positive feelings, for instance stating: “On [the] one hand sadness and emptiness. On the other hand relief, because I associate the local population with the [forest] fires and with environmental problems” (local inhabitant, 51 years). During the interviews, multiple respondents stated that they believe that the traditional farming practices are problematic for fire risk, especially regarding the ignition of new fires, related to the burning of shrublands to enhance grazing potential.

#### Trade-off of different dimensions of land abandonment

**Fig. 4:**
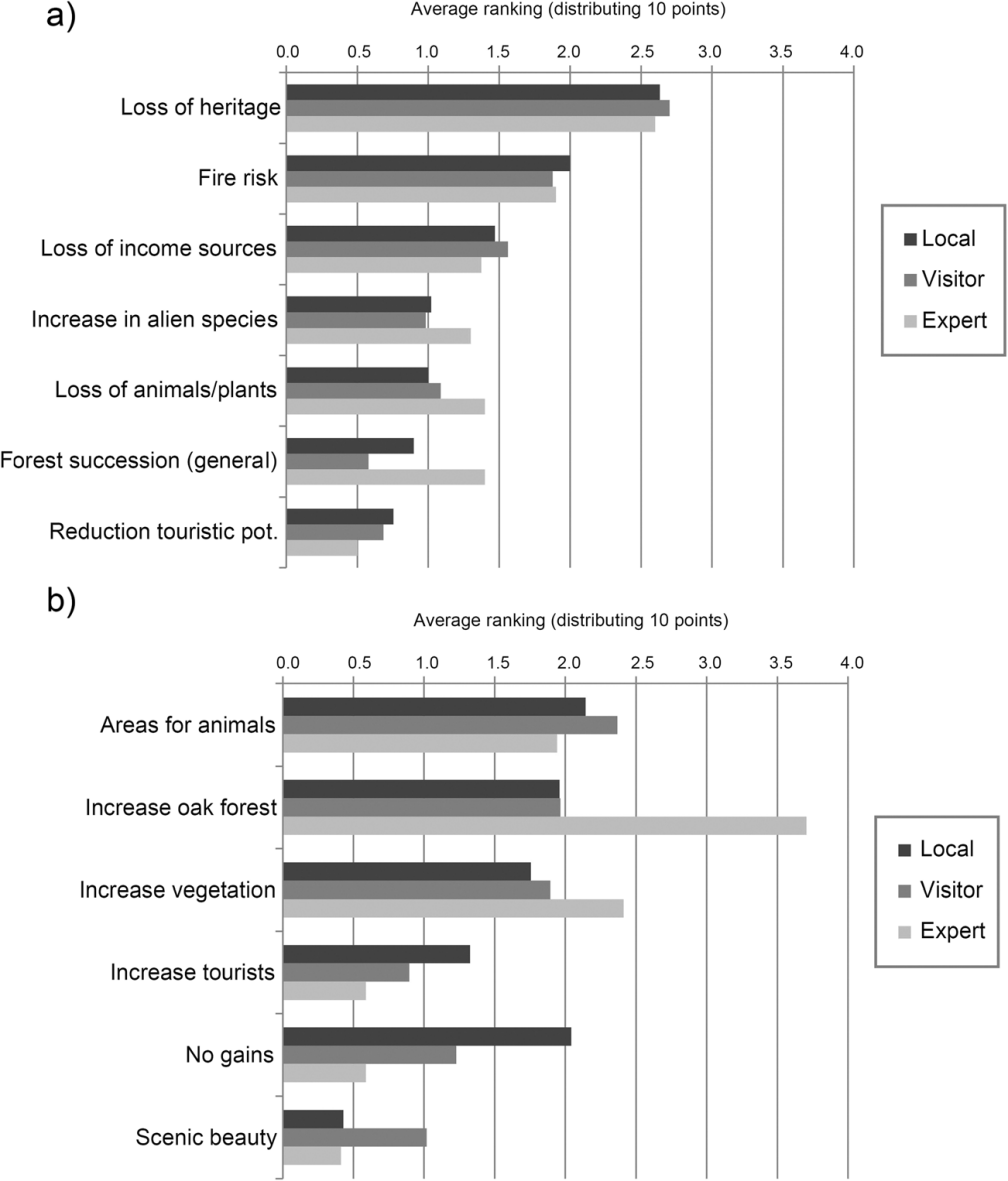
**a** Negative and **b** positive consequences of land abandonment in Castro Laboreiro. Average ranking assigned by respondents; distributing 10 points per respondent

Scores assigned to a selection of consequences and gains of land abandonment revealed that the loss of heritage is regarded as the most important negative consequence, followed by increased fire risk and the loss of income sources (Fig. 4). The second point relates to the fact that forest fires will be more threatening when extensive areas are under succession, although some respondents also linked traditional agricultural practices to increased forest fire risk (see section 3.3.1). The increased area for wild animals and the increase of oak forest are seen as the most important gains. Tourist potential is listed under both the positive and the negative consequences. As a 31-year-old expert commented: “Tourism is slowly starting, but not equal to the loss of agriculture”.

All user groups had remarkable uniform responses to the negative consequences, while for the positive consequences, the increase in oak forest is significantly higher when rated by the experts (χ^2^ = 12.146, d.f. = 2, *p* = 0). Local inhabitants clearly are more negative about the gains of abandonment and give a high value for “no gains”, but this effect was not significant.

### Preference for abandoned landscape types

To assess the preference for the different trajectories of landscape development after abandonment, respondents ranked pictures from different types of abandoned landscapes in Castro Laboreiro based on attractiveness. Overall, oak forest was clearly the most preferred landscape future, followed by low shrubland and tall shrubland. Acacia encroachment was the least preferred, both for the valleys and for the plateau part of the landscape (valley *χ*^2^ = 183.8546, d.f. = 4, *p* = < 2.2 × 10^−16^, plateau *χ*^2^ = 178.1069, d.f. = 3, *p* = < 2.2 × 10^−16^). When evaluating the differences between the valley and the plateau, we find that for the five landscape options in the valley, plantations and oak forest were both preferred the most, with an average of 2.1 versus 2.2, respectively. A more detailed analysis shows that 42 respondents (35%) have oak forest as their first pick for both the valley and the plateau part of the landscape, while 30 (25%) have plantation as their first pick for the valley in combination with oak forest for the plateau.

Although oak forest is clearly the most dominant pick for the plateau, it currently only occurs in a limited number of locations, due to grazing and a limited seedbank. This is confirmed by some of the local respondents. A 58-year-old local inhabitant notes: “There is no oak in the plateau. So tall shrubs and *giestas* [broom] is what we can have and it is beautiful. [Also useful for] firewood”. On the other hand, oak forest is fast developing in the valley area, as a result of limited grazing and agriculture. As noted by a 57-year-old expert: “In the valley, [the] agriculture is marginal. Only hay to feed the cows is viable […] so the oak can easily regenerate in there”.

In terms of the user groups preferences, visitors scored the Acacia encroachment more positive than both the local inhabitants and the experts, with significant results for both the plateau and the valley (plateau *χ*^2^ = 5.5697, d.f. = 2, *p* = 0.01; valley *χ*^2^ = 12.369, d.f. = 2, *p* = 0). An opposite effect is visible for oak forest in the valley. Here, visitors scored the oak forest significantly lower than local inhabitants and experts (*χ*^2^ = 12.369, d.f. = 2, *p* = 0). These results are in line with our expectations, as both local and expert respondents are more familiar with the effect of Acacia encroachment (an invasive species) on the landscape, which quickly became a plague after initial planting.

**Fig. 5:**
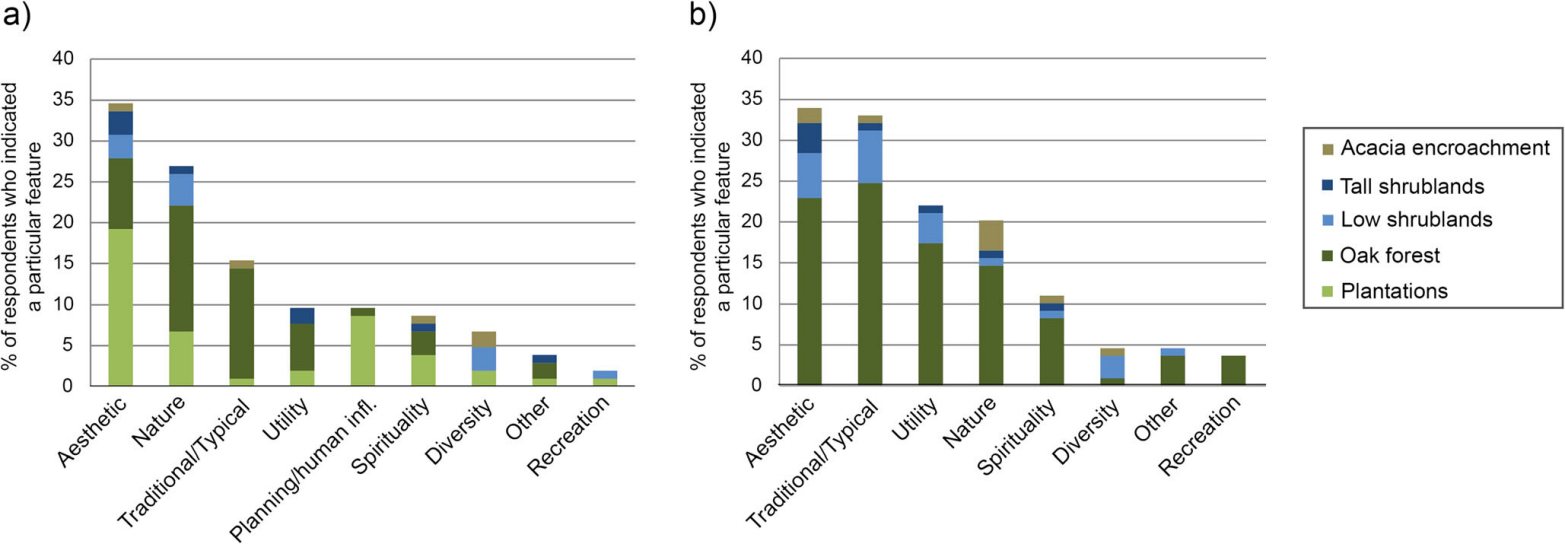
Classified explanations (emotions/reactions) to the preferred abandonment landscapes of the valley (**a**) and the plateau (**b**). Typical responses to this question are available in Online Resource 2

We also collected the reasons for abandonment landscape preferences in an open question (Fig. 5). Aesthetic reasons were most commonly given, followed by reasons based on natural value, utility and being traditional/typical for the area. Overall, oak forest is mainly associated with a positive aesthetic, being a native and symbolic species for the area and having useful benefits. A specific category for the valley emerged on planning/human influence, referring to the structure and human origin of forest plantations. Plantations were perceived positively by the respondents, especially those indicating reasons for their preferences that were related to aesthetics, the organization of the plantations, the human origin and the financial benefit. A local respondent (58 years) for instance stated: “This is income. In the valley it can be a good option for the abandoned fields”.

Utility as positive feature of landscapes is significantly more mentioned by local inhabitants for the valley landscapes (Fisher’s test *p* = 0.01868, for local—visitor). For the plateau section of the landscape, both local respondents and experts mention usefulness as an important feature (Fisher’s test *p* = 0.0246). Here, experts mainly refer to the use of the low shrubs area for grazing. A focus on natural features of the landscape was most dominant in the expert group, followed by visitors and locals, with significant group differences for the valley (Fisher’s test *p* = 0.00436). Other classified features of the abandonment landscapes did not have significant group differences for the user groups.

### Management preferences

To assess possible management directions of Castro Laboreiro, we assessed the preferred overall management direction. When asked about specific landscape management aimed at more farmed areas in the valley, local inhabitants clearly had the highest agreement, followed by visitor and experts (4.5 for locals, 4.0 for visitors and 2.9 for experts on a 5-point Likert scale). The group differences were significant (*χ*^2^ = 18.8017, d.f. = 2, *p* = 0). When the overall preferred management direction was assessed as a semantic differential question from “more traditional farming” to “more natural areas”, both visitors and locals indicated a slight preference for traditional farming (2.5 and 2.9 respectively on a 5-point scale), while experts had a slight preference for more natural areas (3.3 on a 5-point scale), although individual experts were divided on this question. The group differences were significant (χ2 = 6.2087, d.f. = 2, *p* = 0.04, visitor—local *p* = 0.0414). The correlation (Spearman’s rank) between respondent characteristics and the preferred management direction showed weak but significant relationships for several characteristics. Respondents who preferred a management focused on traditional agriculture often have either a higher age, a low income (< 500 €), are from Castro Laboreiro and/or have a connection with agriculture (see Online Resource 2 for more information).

Specific management practices noted as important by respondents included fire risk management (68%), active management for key plants and animals (56%), low intensity grazing (35%) and the development of the wilderness character of the area (32%). Several respondents also mentioned the need for better cleared hiking trails and roads. For specific preferences per user group, see Online Resource 2. Furthermore, the comments on the management showed distrust for the park authority, for instance in handling the fire risk. When discussing the suitable levels of responsibility, about half of the respondents indicated that land management is a multilevel issue with connected responsibilities for park authorities, local governance and the national level. Here, most respondents saw the national level as mainly having a financial role. Regional and European level responsibilities were indicated less (33 and 18%, respectively).

## Discussion

### Attitudes towards land abandonment

Our results show that although land abandonment has been occurring for decades in Castro Laboreiro, the importance of traditional farming is still remarkable, and its cultural values and landscape identity are acknowledged by both the community and its visitors. Despite a clear negative response to abandonment, this cannot safeguard the local traditional livelihoods and cultural values, even when those are considered as assets by a large part of the tourists visiting the region.

A general negative attitude towards land abandonment confirms our hypothesis and is supported by studies in other countries (Benjamin et al. 2007; Höchtl et al. 2005; Hunziker 1995; Ruskule et al. 2013), although several studies that focused on attitudes towards rewilding found more positive attitudes (Bauer et al. 2009; Van den Berg and Koole 2006). The latter are theorized to be influenced by differences in environmental value orientation, with environmentalists expected to have a strong preference for wilderness (Kaltenborn and Bjerke 2002). Based on our regression analysis, we reported that experts and people that do not see human influence as necessary in landscapes are more positive about abandonment. A connection with agriculture was a non-significant factor in the model. Based on other studies, one would expect the opposite effect (e.g. Van den Berg and Koole 2006), but we believe that our results are mainly caused by a high level of agricultural connection throughout the data sample, including the expert group.

As expected in our hypothesis, the assessment of the specific aspects of abandonment revealed that land abandonment is mainly linked to negative emotions. The most dominant emotions (i.e. sadness, feeling left behind and emotions related to the loss of agriculture and tradition) indicate the large attachment that people have with the traditional ways of living and are negatively related to changes affecting this. The responses reflect results from other studies, which report that different aspects linked to abandonment can result in feelings of desolation, isolation, oppression and loss of contact (Bell et al. 2009; Benjamin et al. 2007; Ruskule et al. 2013). For an abandonment study in Latvia, interviews revealed that good management and productive use of land where seen as important for landscape quality by the local inhabitants (Ruskule et al. 2013). This was also reflected in our study. For instance, when discussing measures to improve the local conditions, most respondents mentioned “cleaning up” the fields. Previous studies also report that farmers have a strong aesthetic preference for managed landscapes (see, e.g. Burton 2012).

We hypothesized that visitors would be less negative to abandonment, both in general responses as in emotions, as other studies have reported positive or mixed responses of tourists for a more wild landscape (Höchtl et al. 2005; Van den Berg and Koole 2006). However, this hypothesis was not confirmed and differences between the locals and visitors of the area were relatively small. Possible explanations are the large percentage of the visitors with an agricultural background and the fundamental role of agricultural activity in the social construction of the rural landscape in Portugal (Figueiredo 2008). A third explanation was revealed through the in-depth answers of the tourists, which often included the elements “nostalgia”, “loss of traditions” and loss of heritage. This focus reflects current rural tourism, which is often characterized by focus on “return to the origins” and nostalgia for the “good old days” (see e.g. Kastenholz et al. 2012). Previous exploratory studies on the “sense of place”, i.e. an umbrella concept encompassing place relations such as place attachment, of visitors in contrast with local inhabitants in mountainous areas have shown that both groups focus on similar place characteristics but attach different meanings and significance to these characteristics (Kaltenborn and Williams 2002; Kianicka et al. 2006).

The importance of cultural heritage is even more apparent for local inhabitants. From our results, it is clear that the local inhabitants view traditional agriculture as a clear part of their identity and resist changes related to abandonment. This resistance can be viewed as a reaction to the changing role of the rural zone, which transformed from a place of agricultural production (“productivism”) into a leisure and experimentation space with a multifunctional focus (“post-productivism”), in which new narratives developed, often influenced by an urban population, defining what it is to be rural and authentic (López-i-Gelats et al. 2009). This has resulted in controversies and oppositions within local populations of many rural regions (del Mármol and Vaccaro 2015). Carolino (2010) underline that local sentiments often “go beyond mere nostalgic resistance to change” as land use and farming have a clear function for social cohesion, which is, e.g. embedded in the vision of what it entails to be a villager.

### Management of abandonment landscapes and future pathways

The preferred future management direction of the abandonment areas in Castro Laboreiro by local inhabitants and visitors is one of traditional farming, while experts are more focused on the development of natural areas. This attitude contrasts with the current policies in the area that focus mainly on abandonment prevention, e.g. through subsidies for certain agricultural management practices through the Integrated Territorial Interventions (ITI), administered by the rural development program of Portugal (Beilin et al. 2014; van Berkel et al. 2011). Our results deviate from our initial hypothesis that was based on previous studies that show a general negative response towards spontaneous abandonment (Soliva et al. 2008) and a clear preference for a return to the traditional cultural landscape by experts and decision makers (e.g. for a case study in Switzerland; Hunziker et al. 2008).

An important addition of our study to the existing literature on land abandonment is that we assess not only the process of abandonment, but also the different stages and possible outcomes. We found that these different outcomes clearly yielded different responses and preferences. The use of field photographs of the different post-abandonment landscapes did not allow disentangling which landscape features determined the preferences. Landscape features such as the degree of naturalness, openness and landscape variety can influence the perceived visual beauty of landscapes (see Tveit et al. 2006 for an overview). Nevertheless, the mixed approach of photograph ranking, landscape explanations/associations and user group analysis provided important inputs for local policy and future possible management directions of the landscape in Castro Laboreiro.

Inclusion of the perceptions of different societal groups in landscape planning is important in two ways: in the identification of planning goals that reconcile the views of various public groups and in reducing conflicts (Hunziker et al. 2008; Smith et al. 2011). Our elicitation of landscape preferences associated with the different abandonment landscapes can have an important function in the first goal by finding a common ground for landscape management of these novel landscapes. For instance, while the utilitarian focus of local inhabitants in Castro Laboreiro can be a source of conflict, an acknowledgement of this perspective can help in the acceptance of new landscape management practices, e.g. management targeted at oak forest, which is associated with useful and symbolic features by local inhabitants and highly rated by experts. Furthermore, a reconciliation of agricultural management linked with conservation goals is a policy measure that could bridge conflicting aims.

The second goal regarding conflict reduction is also important, as local inhabitants in Portugal are often excluded from the formation and management of rural policies and planning leading to management conflicts and disrespect of regulations (Figueiredo 2008). For Castro Laboreiro specifically, this means the reconciliation of the views of different user groups with the stipulations and regulations of the National Park, which currently has considerable planning power in the area. Our interviews have shown that different views on landscape management have led to a distrust by local inhabitants of the park authority and others that have focused on ecological development. These conflicts were also reported in a previous study in Castro Laboreiro (see van Berkel et al. 2011).

### Implications for management

Rewilding of oak forests is indicated as the most preferred outcome for the plateau while both oak forest and forest plantations gain the highest scores in the valley region. These results can be used to inform future management directions, including the implementation of active management to steer the land abandonment transitions away from threats such as forest fire risk. In a more spatially targeted and nuanced approach, rewilding could be used as a management option for certain areas, while other locations would benefit from different management and functions. In line with our results, this would for instance result in rewilding focus targeting oak forest regeneration in the valley, while in the plateau, active management of oak forest would be required to cater for a limited seedbank and preferred residential and agricultural activities. For such a management focus, explicit acknowledgement of the different needs of user groups and consensus-oriented stakeholder involvement is essential.

Areas in transition such as Castro Laboreiro can undergo several pathways, ranging from a (social) collapse, to an agricultural focus purely dependent on subsidies, to a focus on new ways to reconnect with the global economy, e.g. by finding entrances to a niche market (Vaccaro 2010). Interviews with experts and stakeholders in Castro Laboreiro gave some insights in the local barriers and frictions for rural development in Castro Laboreiro. Landscape abandonment is seen by these actors as a major threat to the regional character of the area, which they fear will negatively influence the area as a tourism asset with ability to attract more visitors. Challenges to rural and tourism development, however, include a lack of entrepreneurial collaboration and a focus on agricultural development projects only. This has, for instance, resulted in the failure to maintain several initiatives (e.g. branding of local agricultural products). Based on the diversity of responses and the differential potentials between different parts of the landscape, we propose spatially targeted and nuanced management of abandoned areas. This could include both a conservation and rural development focus, by combining an active landscape management of abandoned areas with diversification options for agricultural areas, for instance by the stimulation of initiatives regarding ecotourism or rural tourism.

## Electronic supplementary material

ESM 1 (DOCX 24222 kb)

ESM 2 (DOCX 37 kb)
