# Supplementary material for: Abandonment landscapes: user attitudes, alternative futures and land management in Castro Laboreiro, Portugal
Source: Reg Environ Change. 2018 Feb 10;18(5):1509–20. doi: 10.1007/s10113-018-1294-x (PMC6448354; doi:10.1007/s10113-018-1294-x)
Supplement: Supplementary file 1 — (DOCX 24222 kb) [file 10113_2018_1294_MOESM1_ESM.docx]

**Online resource 1**

Abandonment landscapes: user attitudes, alternative futures and land management in Castro Laboreiro, Portugal

Emma H. van der Zanden*, Sónia M. Carvalho-Ribeiro, Peter H. Verburg

*Environmental Geography group, VU University Amsterdam, email: emma.vander.zanden@vu.nl

**
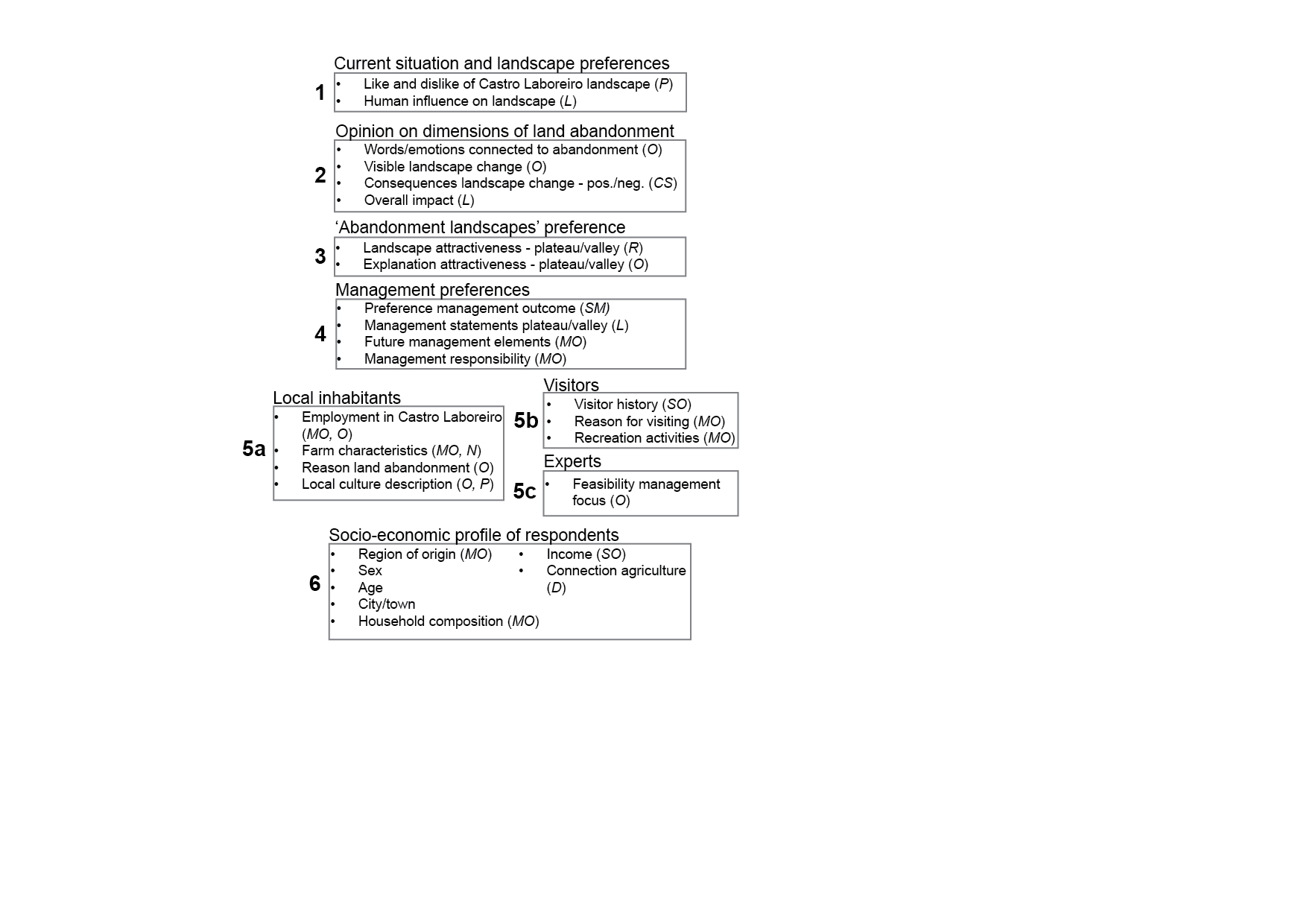
Figure 1:** Content of the questionnaire, with specification per question type.

*P = visual description, L = statement with Likert-scale, O = open question, CS = Constant Sum question, R = ranking question, SM = Semantic differential statement, MO = Multiple option question, SO = Single option question = D = dichotomous question.*

**Sample-selection criteria per user group**

For local inhabitants, the criteria was to be an inhabitant or home owner in Castro Laboreiro, with a sampling focus to include all age groups, levels of education, both sexes and different levels of engagement in agriculture. For tourists, the selection criteria was to be a visitor in Castro Laboreiro and we focused to include all age groups, different focuses of the recreational activities, places of residence, level of education and both sexes. For the expert group, we selected experts and decision makers that worked on landscape management issues on a regular basis: in policy, education, research or management. The requirement for this group was to be familiar with the Castro Laboreiro and Peneda-Gerês National Park.

**Table 1:** Different abandonment landscapes, as delineated in Castro Laboreiro, with their main species composition.

| *Abandonment landscape* | *Main species* | *Location* |
| --- | --- | --- |
| Oak forest | Quercus robur and/or Quercus pyrenaica. | Valley - Plateau |
| Tall shrublands | Dominated by different broom species, such as *Cytisus scoparius, Cytisus grandiflorus DC*. | Valley - Plateau |
| Low shrublands | Mainly perennial herbs and woody species. | Valley - Plateau |
| Acacia encroachment | Mainly *Acacia dealbata* | Valley - Plateau |
| Plantations | Both *Pinus pinaster* and *Pinus sylvestris* as well as subsidized fruit trees such as chestnut. | Valley |

**Figure 2:** Different abandonment landscapes, as delineated in Castro Laboreiro. The photographs on the left show close up views and the photos on the right are distant views. This set of photographs represents the landscapes of the valley section at different stages of regrowth after agricultural abandonment (see Figure 3 for the photographs representing the plateau section)


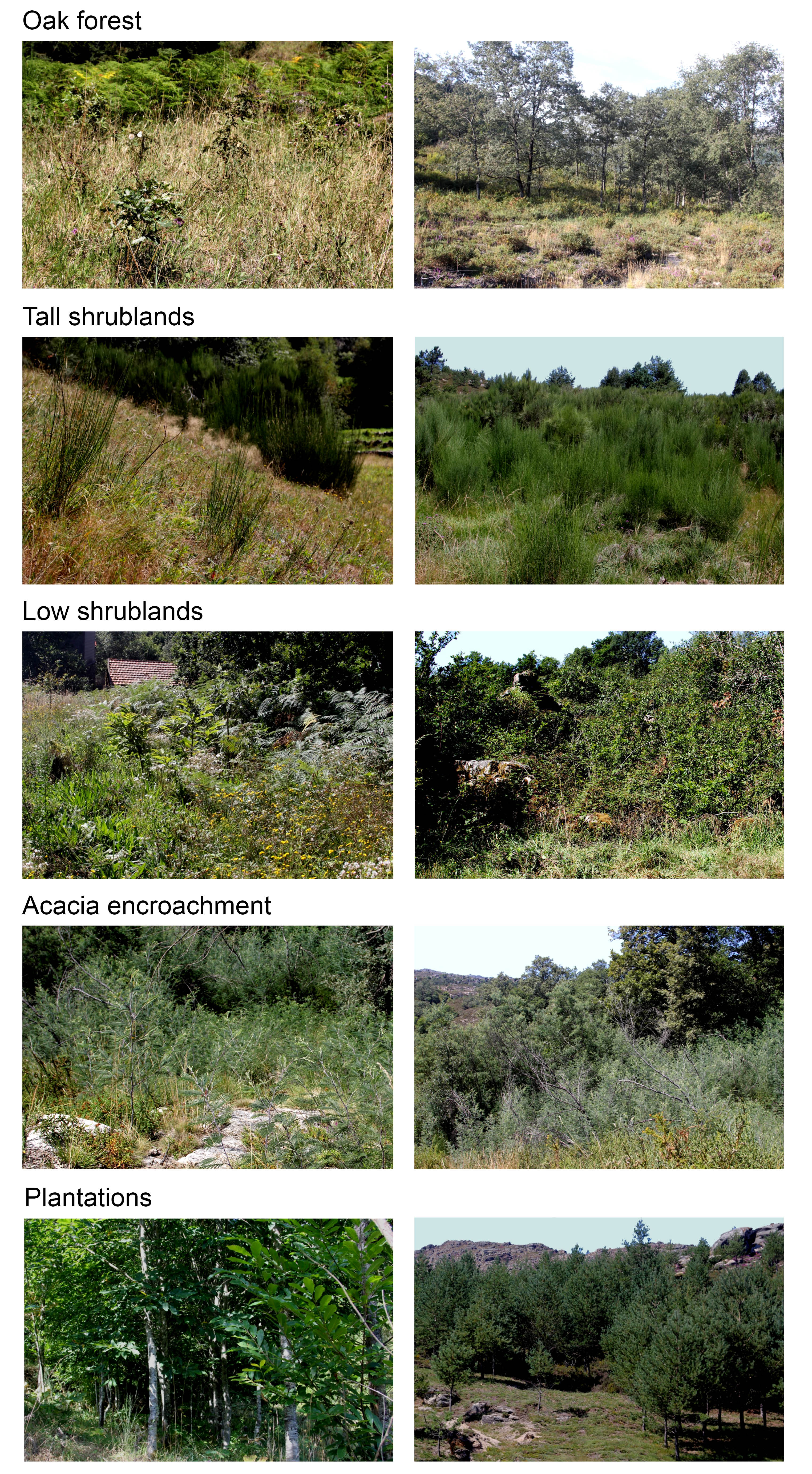


**
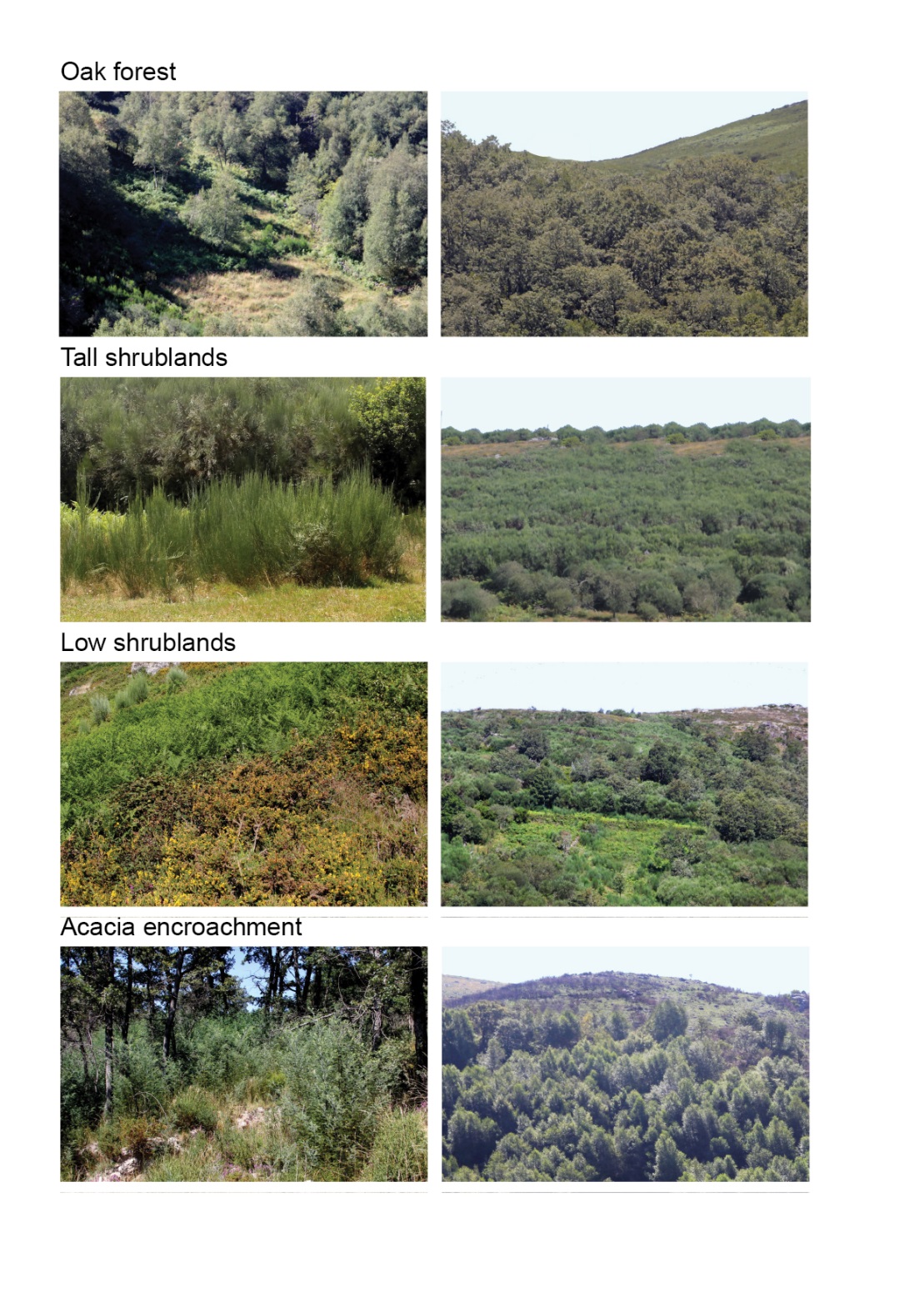
Figure 3:** Different abandonment landscapes, as delineated in Castro Laboreiro. This set of photographs represents the landscapes in the plateau section of the landscape at different stages of regrowth after agricultural abandonment
