# Supplementary material for: Abandonment landscapes: user attitudes, alternative futures and land management in Castro Laboreiro, Portugal
Source: Reg Environ Change. 2018 Feb 10;18(5):1509–20. doi: 10.1007/s10113-018-1294-x (PMC6448354; doi:10.1007/s10113-018-1294-x)
Supplement: Supplementary file 2 — (DOCX 37 kb) [file 10113_2018_1294_MOESM2_ESM.docx]

**Online resource 2**

Abandonment landscapes: user attitudes, alternative futures and land management in Castro Laboreiro, Portugal

Emma H. van der Zanden*, Sónia M. Carvalho-Ribeiro, Peter H. Verburg

*Environmental Geography group, VU University Amsterdam, email: emma.vander.zanden@vu.nl

**Table 1:** Descriptive summary of the interviewees. Significant differences for all respondents or of a user group with both other user groups indicated by **, significant difference between selected user groups are indicated by *.

|  |  | *All Respondents* | *Locals* | *Visitors* | *Expert* |
| --- | --- | --- | --- | --- | --- |
| *n* |  | 122 | 49 | 57 | 17 |
| Age (average)^1^ |  | 47** | 53** | 43 | 43 |
| Gender (%) | male | 43 | 41 | 47 | 35 |
|  | female | 57 | 59 | 53 | 59 |
| Education (%) | basic^2^ | 11** | 20* | 7 | 0 |
|  | intermediate | 27 | 33 | 28 | 6 |
|  | high^3^ | 49** | 27** | 56** | 88** |
|  | retired | 12 | 20 | 9 | 0 |
| Household membership (%) | single | 31 | 27 | 39 | 18 |
|  | partner^4^ | 25** | 35 | 14 | 29 |
|  | partner + kids | 36 | 31 | 37 | 47 |
|  | kids^#^ | 4 | 6 | 4 | 0 |
|  | family^#^ | 2 | 0 | 5 | 0 |
| Income (%) | <500euro | 21** | 33* | 18 | 0* |
|  | 500-1000 euro | 17 | 24 | 16 | 0 |
|  | 1000-1500 euro | 16 | 14 | 14 | 24 |
|  | 1500-2500 euro^5^ | 20** | 8** | 25 | 35 |
|  | >2500 euro^6^ | 21** | 10* | 28* | 29 |
| Location of  origin (%) | Castro Laboreiro^7^ | 38** | 78** | 14 | 0 |
|  | North of Portugal^8^ | 11** | 2** | 14 | 24 |
|  | Central and Southern Portugal^9^ | 36** | 20** | 44 | 53 |
|  | Abroad^10^ | 13** | 0* | 25* | 12 |
| Connection w. agriculture^11^  Degree of human influence (rating^12^)^13^ | | 67**  2.03** | 88**  2.58* | 54  1.72 | 47  1.75 |

^1^χ^2^ = 11.254, d.f. = 2, p = 0.0036.^2^Fisher’s exact test *p* = 0.0296, local – visitor *p* = 0.04939. ^3^Fisher’s exact test *p* = 2.176e-06. ^4^Fisher’s exact test *p* = 0.03583.^#^ not included ^5^ Fisher’s exact test *p* = 0.01019, local – expert *p* = 0.006869. ^5^ Fisher’s exact test *p* = 0.01303. ^6^Fisher’s exact test *p* = 0.03646, local – visitor *p* = 0.02771. ^7^Fisher’s exact test *p* = 4.643e-14. ^8^Fisher’s exact test *p* = 0.00969. ^9^Fisher’s exact test *p* = 0.007091. ^10^Fisher’s exact test *p* = 0.0001538, local – visitor *p* = 7.918e-05^11^Fisher’s exact test *p* =5.759e-05.^12^Rating of statement ‘Natural areas without human influence are useless’ on 5-point Likert scale, 1 = not agree at all, 5 = fully agree ^13^χ2 = 6.515, d.f. = 2, p = 0.04.

**Table 2:** Linear regression for overall abandonment impact score.

|  | *Coefficient* | *Standard deviation* | *t value* | *p>\|t\|* |
| --- | --- | --- | --- | --- |
| Intercept | 2.436 | 0.20966 | 11.617 | <2*10^-16^*** |
| Expert group | 0.602 | 0.29216 | 2.059 | 0.0419* |
| Human influence | -0.135 | 0.06706 | -2.006 | 0.0474* |
| Local | -0.292 | 0.22972 | -1.269 | 0.207 |
| Connection agriculture | 0.096 | 0.23886 | 0.402 | 0.6884 |
| Significance levels: 0 = ***, 0.001 = **, 0.01 *  d.f. = 107  AIC = 321.52, R^2^ = 0.13, Adjusted R^2^ = 0.10 | | | | |

**Table 3:** First ranked abandonment landscape pictures and typical explanations (emotions/reactions) to the landscapes, divided by classes delineated from the content.

|  |  | *Oak forest* | *Plantations* | *Low shrublands* | *Tall shrublands* | *Acacia encroachment* |
| --- | --- | --- | --- | --- | --- | --- |
| Valley | *n* | 51 | 46 | 12 | 7 | 5 |
| Plateau | *n* | 81 |  | 20 | 10 | 10 |
|  | Aesthetic | -(Natural) beauty  -Beautiful in spring  -Type of trees  -Different colors  -Beautiful leaves | -Beautiful  -Type of trees (pine/tall)  -Healthy appearance  -Scenic beauty  -Green | -Different colors of flowers  -Beautiful in spring  -Aesthetic reasons  -Attractive  -Green | -Beautiful flowers in spring  -Green  -General overview  -Type of plant  -Beautiful | -Green |
|  | Nature | -Good for ecological system  -Wild  -Nature getting back  -Natural richness  -Allows animals and undergrowth | -Feeling of wilderness  -Variety of trees  -Maintaining species  -Natural | -Natural | -Important refuge for animals | -Fresh air |
|  | Utility | -Good quality timber  -Source of firewood  -Shade  -Food for bees  -Fire protection  -Refreshes air | -Shade  -Income/money | -Grazing area  -Good honey | -Source of firewood  -Wood |  |
|  | Traditional/Typical | -Native tree  -Most predominant tree  -Part of natural richness of the region  -Iconic/Symbolic of the region  -Contributes to Castro culture  -Traditional  -Part of Northern Portuguese landscape | -Traditional | -Typical for plateau |  |  |
|  | Spirituality | -Tranquility  -Life  -Freedom  -Sacred  -Joy  -Internal peace  -Magic | -Fertility  -Mystic area  -Freedom  -Tranquility | -Joy | -Different spiritual experience on the plateau (fits with the *giesta*) | -Peace |
|  | Order/Planning |  | -Planning  -Organized  -Human involvement  -Order |  |  |  |

**Table 4:** Spearman’s rank correlation the preferred management direction (high values of management direction indicate management focused on natural areas, low values indicate management focused more on traditional agriculture) and respondent characteristics.

|  | *Spearman’s Rho* | |
| --- | --- | --- |
| Human influence | -0.31*** | |
| Age | -0.20**∙** | |
| Basic education  Higher education | -0.29***  0.31*** | |
| Income < 500 euro | -0.31*** | |
| Income between 1500 and 2500 euro | 0.20**∙** | |
| From Castro Laboreiro | - 0.34*** | |
| From Central and Southern Portugal | 0.27*** | |
| Connection agriculture | - 0.18**∙** | |
| Significance levels: ‘**∙**'<0.05 ‘*'<0.01, '**'<0.001, '***'< 0.0001 | |  |

**Figure 1:** Preferences for specific future management practices in Castro Laboreiro (% of user groups who indicated a particular feature)
